# A Case of Psychosis in Disulfiram Treatment for Alcoholism

**DOI:** 10.1155/2014/561092

**Published:** 2014-04-10

**Authors:** Raquel Calvão de Melo, Rui Lopes, José Carlos Alves

**Affiliations:** ^1^Department of Psychiatry, Divino Espírito Santo Hospital, Azores, 9500-370 Ponta Delgada, Portugal; ^2^Clinic of Psychiatry and Mental Health, São João Hospital, 4200-319 Porto, Portugal

## Abstract

*Background*. Disulfiram, a drug used in the treatment of alcohol dependence, is an inhibitor of dopamine-**β**-hydroxylase causing an increase in the concentration of dopamine in the mesolimbic system. In addition to the physical symptoms associated with concomitant use of alcohol, disulfiram may lead to adverse events, when used alone, including psychosis. *Aims*. To report a case of a rare complication when using disulfiram for alcoholism treatment in a patient in alcoholic abstinence. *Case Report*. We describe the case of a 42-year-old male patient, who developed psychotic symptoms 3 weeks after initiating treatment with disulfiram for alcohol dependency. The patient had a history of chronic alcoholism for 12 years and was under disulfiram treatment (250 mg/day) for 1 month, with no other past history of psychiatric illness. The symptoms worsened after he initiated alcohol consumption, while taking disulfiram. The patient was hospitalized and disulfiram was suspended. After 4 days he was asymptomatic and at 6-week follow-up remained asymptomatic. *Conclusion*. Treatment with disulfiram can lead to the appearance of psychosis in patients with increased vulnerability. In clinical practice, psychosis in the context of alcoholism with disulfiram therapy is often neglected and should be taken into account.

## 1. Background

Disulfiram (tetraethylthiuram disulfide) is a quaternary ammonium compound, which is as an agent used for treating alcohol dependency for 60 years [1, 2].

It was synthetized for the first time in 1881 and was used in industry as catalytic accelerator for the vulcanization of rubber. Later in 1910, workers of that firm showed aversive reactions after drinking alcohol at lunch, but it was only in 1937 that the potential of disulfiram in alcohol dependency was discovered [3].

Hald and Jacobson, two Danish physicians, in 1940, became ill while taking disulfiram as an anthelmintic, after alcohol consumption [4], and in 1948 it started being used for alcohol dependency [3].

It is an inhibitor of dopamine-*β*-hydroxylase causing an increase in the concentration of dopamine in the mesolimbic system. In addition to the physical symptoms associated with concomitant use of alcohol, disulfiram may lead to adverse reactions when used alone, including psychosis [4]. So it is not awkward to imagine that when dopamine concentrations rise, it creates the conditions for developing psychotic symptoms.

Initially, when disulfiram started being used, the doses were much higher (1000–3000) than today (250 mg–500 mg) [5] resulting in more reports of toxicity, delirium, and psychosis than nowadays.

Also, in patients with a family history of psychosis, disulfiram induced psychosis is more likely due to genetic background [6].

## 2. Case Report

A 34-year-old male patient, married, living with his wife and two daughters, working as an electrician, presented as an acute emergency case with a 5-day history of paranoid psychosis.

He was convinced that there were cameras and someone was watching him, would not eat without his wife trying first the food, had aggressive behavior towards his wife, described “voices inside his head,” and had total insomnia for 3 days. He was taken to the emergency room after being found by his friends “saying things with no sense” near his house. Prior to that day he had been drinking. His wife described that these symptoms started 5 days before he was admitted in the ER and that at that time the patient was in alcoholic abstinence.

Our patient had been abusing alcohol for almost 12 years. One month before the beginning of symptoms he was observed in a psychiatry consultation, in which he was diagnosed with alcoholic dependency, with no psychotic symptoms, and started treatment for alcoholic dependency with disulfiram. The patient was abstinent since he started the treatment and according to his wife he started drinking after the psychotic symptoms appeared, with worsening of symptoms after that.

Besides disulfiram he was also taking acamprosate 666 mg/day and topiramate 200 mg/day. His motivation for being abstinent was the conflicts he was having with his wife due to his alcohol dependence.

The patient had no history of delirium tremens, seizures or alcoholic hallucinosis, or other psychiatric illness. He also had no other relevant medical history. In his family history he had his father who was diagnosed with chronic alcoholism and presented psychotic symptoms during a short period of time, when he had a heavier alcoholic consumption.

At mental state examination he was poorly cooperative, had anxious humor and nonspontaneous incoherent speech, and had delusions of persecutory type and verbal hallucinations; he was very suspicious and agitated and had no insight into his medical condition.

When he was admitted at the emergency room he also presented symptoms of alcohol disulfiram reaction with tachycardia, sweating, and vomiting.

Serological examination showed hepatic alterations (GGT-124, ALT-92); alcohol was positive—0.074, and urine drug test was negative. Also he performed a CT scan that showed no alterations.

The patient was admitted in our inpatient facilities; disulfiram was suspended and risperidone 6 mg/day, quetiapine 100 mg/day, and lorazepam 2.5 mg/day were started.

His paranoid symptoms remained for 4 days and at the 5th day of inpatient the symptoms disappeared completely and he became approachable and unsuspicious. For 15 days we slowly reduced the patient's medication, and by the time he was discharged he was only taking benzodiazepines.

At the 6-week follow-up in outpatient consultation, he was asymptomatic, without alcohol consumption, and had returned to his work. In this observation he started again topiramate 200 mg/day and acamprosate 666 mg/day and nowadays remains asymptomatic.

## 3. Discussion

Disulfiram acts as a deterrent against drinking [2], is used to manage the impulsive drive to drink in patients with alcohol dependency, and is recognized for its physical and psychological effects [7]. However, it is estimated that 25–75% of patients under disulfiram treatment have concomitant alcohol consumption [8].

In the presented case, our patient was able to maintain abstinence for almost a month, having relapsed into drinking after the psychotic symptoms appeared.

When there is concomitant alcohol consumption, it produces sensitivity to alcohol, leading to an unpleasant reaction, ethanol-disulfiram reaction, caused by the accumulation of acetaldehyde [9] through the inhibition of the enzyme acetaldehyde dehydrogenase with histamine release [10]. Our patient also presented physical symptoms of this reaction, when he started drinking.

The most common symptoms of this adverse reaction are hypotension, tachycardia, diaphoresis, flushed face, headache, nausea, vomiting, and confusion; however, only 28% of patients that are under disulfiram treatment experience these symptoms, after having alcoholic consumption [11, 12].

Disulfiram can also cause adverse symptoms, when used alone, such as drowsiness, fatigue, impotence, headaches, skin eruptions, neurological toxicity, and psychosis [10].

In our case report, we could wonder about the etiology of psychotic symptoms. Could it be a bipolar disorder with a manic episode, symptoms of alcohol withdrawal, or even abuse of other substances?

In our case the patient presented with an anxious mood, not elevated mood; although the craving for alcohol drinks and insomnia could lead us to a possible manic episode, there was no previous history of mood disorders, and when he was observed in psychiatric outpatient consultation, he had euthymic mood and at that time did not presented psychotic symptoms. Also he had no family history of mood disorders. The other possible diagnosis that we could think of would be alcohol withdrawal; however, if this was the case symptoms usually would occur within 8 hours after the last drink and they reach the peak by 24–72 hours. Symptoms can include seizures, hallucinosis, and delirium tremens and usually last 1 week. Our patient was abstinent for almost 1 month when the symptoms started, so alcohol withdrawal would not be the most probable diagnosis.

Psychotic symptoms can occur related to alcohol dependency as in intoxication, withdrawal, alcohol-induced psychotic disorder, and delirium. In alcohol-induced psychotic disorder, symptoms, such as delusions and auditory and visual hallucinations, occur along with those usually associated with alcohol intoxication or withdrawal [13].

We can also exclude symptoms caused by abuse of illicit drugs because the urine drug test was negative. So the most probable diagnosis would be psychosis caused by disulfiram treatment.

Disulfiram's major metabolites, carbon disulfide and diethyldithiocarbamate (DDC), are associated with side effects [11]. DDC may cause the inhibition of dopamine-*β*-hydroxylase leading to an increase of dopamine concentration in mesolimbic system, inducing psychosis [14] as stated by the “dopamine hypothesis” described in schizophrenia [4].

Norepinephrine synthesis and production are also blocked by disulfiram, which may relate to psychosis and mania while taking disulfiram, because dopamine-*β*-hydroxylase is blocked, inhibiting the turnover of dopamine into norepinephrine leading to the increasing of dopamine [6].

Dopamine-**β**-hydroxylase increases with stress to enhance the turnover of noradrenaline during stress. When this enzyme is inhibited due to disulfiram treatment, the adaptive response to stress is compromised, and the susceptibility to dopamine increase is higher. Psychosis is more likely in patients with low dopamine-*β*-hydroxylase concentrations [15].

Abstinence from alcohol is associated with higher levels of anxiety, emotional distress, and alcohol craving, because of the disruption in normal functioning of the peripheral stress pathways, namely, the HPA axis and the autonomic components. These pathways are involved in physiological regulation of the stress response [16].

In our case, the patient was abstinent for almost a month, causing an increase of stress that could have triggered the development of symptoms. Because of his family history, he could have low levels of dopamine-*β*-hydroxylase; so, when it gets blocked by disulfiram, there is a greater predisposition to the development of psychosis.

Patients with family history of psychosis are more vulnerable to precipitants of psychosis than the other patients and general population [17]. So, alcohol addicts with family history of psychosis are more vulnerable to disulfiram induced psychosis due to genetic predisposition [6]; as in our case, he had his father diagnosed with chronic alcoholism at a period where he presented with psychotic symptoms.

Also manic episodes and psychosis have been described in patients with psychiatric comorbidities [18].

Some studies on disulfiram have been developed, one of them by Liddon and Satran, and they concluded that, in 52 patients described to have disulfiram psychosis, actually only five were in fact psychotic and the others had toxic delirium [19].

Krishna et al., in two studies, reported 5 cases of psychosis out of 53 and 6 cases in a total of 52 patients under disulfiram treatment.

Also neuropsychiatric symptoms can appear when patients start alcoholic consumption while taking disulfiram. Toxicity, as in psychotic reactions, appears to be dose related, with the dosages used today being relatively safe to most patients [5].

Adverse reactions can last for 2 weeks after taking disulfiram, but usually they last no longer than 3 days after suspending this drug [20, 21].

## 4. Conclusion

Disulfiram, used for alcoholism treatment, nowadays with lower dosage, 250–500 mg, is an effective drug that conditions patient's behavior towards drinking. However, because of its adverse reactions, namely, ethanol-disulfiram, it has to be prescribed with caution and always under medical supervision.

Literature suggests that disulfiram can cause and exacerbate psychiatric syndromes, namely, psychosis. Nowadays disulfiram is used with some reluctance because of its adverse effects; however, supervised disulfiram treatment in patients with alcoholism can have positive results.

In our case the patient developed psychotic symptoms probably due to his genetic predisposition. This case report highlights the relevance of a careful history of patient's symptoms and family history that should be performed before starting treatment with disulfiram. The psychotic symptoms may be related to dose and duration of treatment, but it still needs more studies to be proven. Careful information should be given to patients under disulfiram treatment.
